# Across-proteome modeling of dimer structures for the bottom-up assembly of protein-protein interaction networks

**DOI:** 10.1186/s12859-017-1675-z

**Published:** 2017-05-12

**Authors:** Surabhi Maheshwari, Michal Brylinski

**Affiliations:** 1 Department of Biological Sciences, Louisiana State University, Baton Rouge, LA USA; 2 Center for Computation & Technology, Louisiana State University, Baton Rouge, LA USA

**Keywords:** Protein-protein interactions, Protein docking, Structural bioinformatics, Machine learning, Gene Ontology filters, *e*FindSite^PPI^, *e*Rank^PPI^

## Abstract

**Background:**

Deciphering complete networks of interactions between proteins is the key to comprehend cellular regulatory mechanisms. A significant effort has been devoted to expanding the coverage of the proteome-wide interaction space at molecular level. Although a growing body of research shows that protein docking can, in principle, be used to predict biologically relevant interactions, the accuracy of the across-proteome identification of interacting partners and the selection of near-native complex structures still need to be improved.

**Results:**

In this study, we developed a new method to discover and model protein interactions employing an exhaustive all-to-all docking strategy. This approach integrates molecular modeling, structural bioinformatics, machine learning, and functional annotation filters in order to provide interaction data for the bottom-up assembly of protein interaction networks. Encouragingly, the success rates for dimer modeling is 57.5 and 48.7% when experimental and computer-generated monomer structures are employed, respectively. Further, our protocol correctly identifies 81% of protein-protein interactions at the expense of only 19% false positive rate. As a proof of concept, 61,913 protein-protein interactions were confidently predicted and modeled for the proteome of *E. coli*. Finally, we validated our method against the human immune disease pathway.

**Conclusions:**

Protein docking supported by evolutionary restraints and machine learning can be used to reliably identify and model biologically relevant protein assemblies at the proteome scale. Moreover, the accuracy of the identification of protein-protein interactions is improved by considering only those protein pairs co-localized in the same cellular compartment and involved in the same biological process. The modeling protocol described in this communication can be applied to detect protein-protein interactions in other organisms and pathways as well as to construct dimer structures and estimate the confidence of protein interactions experimentally identified with high-throughput techniques.

**Electronic supplementary material:**

The online version of this article (doi:10.1186/s12859-017-1675-z) contains supplementary material, which is available to authorized users.

## Background

Protein-protein interactions (PPIs) are ubiquitous and play crucial roles in all biological processes within and between cells by mediating signaling pathways in cellular networks and controlling intracellular communication [1]. Since complex biological systems are governed by sophisticated networks of PPIs, associations between proteins ultimately determine the behavior of the cell. Genome-sequencing projects provide comprehensive datasets of biological sequences and numerous post-genomic projects are largely focused on the exploration and analysis of PPIs across proteomes [2, 3]. The number of possible PPIs in an organism can be scaled as the square of the total number of monomeric proteins, yielding an estimated number of disparate protein complexes in the order of millions. High-throughput approaches allow the large-scale detection of protein-interaction partners in many organisms. Although the PPI data is being produced at a swift pace, the major issues in using the current genome-wide PPI data are a low coverage and high false positive rates [4, 5]. Moreover, inter-study discrepancies between different experimental approaches applied to the same biological system are not uncommon [6]. Last but not least, while these high-throughput methods identify proteins interacting with one another, they do not provide structural information on biologically relevant protein complexes.

On the other hand, interaction details, which can only be obtained from three-dimensional structures, are crucial to fully comprehend interaction mechanisms at the atomic level. Unfortunately, despite ongoing efforts in structural genomics projects to determine complex structures, structural biology is lagging behind in the current trends of high-throughput methods. While the repertoire of monomeric protein structures solved by X-ray crystallography and NMR spectroscopy is increasing exponentially, the structural space of interacting proteins is still far from complete. In fact, there is an increasing gap between the number of identified interactions and the number of 3D structures of these associations. Thus, it is imperative to develop and continuously improve computational techniques to accurately identify interacting proteins and the corresponding complex structures.

A number of computational approaches have been developed to discover and model new interactions at a system level. Modeling complex structures can be accomplished using two distinct types of techniques, template-free and template-based. The former methods, also known as protein docking, construct a complex model by assembling the monomeric structures of target proteins through a conformational search followed by the selection of high scoring binding orientations. In contrast, template-based approaches build complex structures by mapping monomeric targets to experimentally solved template complexes often followed by the refinement of the initial structural framework. Both methods have advantages and disadvantages. Template-based approaches can construct dimeric models directly from target sequences, therefore, monomer structures may not be required. Further, these techniques select templates based on sequence [7, 8], sequence-to-structure [9] and structure alignments [10, 11] often yielding more accurate results than template-free docking [12, 13]. Although dimer templates are available in the Protein Data Bank (PDB) [14] to model all complexes in which the monomer structures are either known or can independently be modeled [15], the success rate of template-based docking is only about 23% when no closely homologous templates with a sequence identity to the target of >40% can be found for at least one monomer chain. Analogous interaction templates cannot be identified in the current PDB to effectively guide template-based docking in those failed cases [16]. The fact that suitable templates are available only for a limited number of interactions significantly lowers the coverage of proteome-scale datasets.

In contrast, template-free methods are, in principle, applicable to those protein targets whose monomer structures are either solved experimentally or can be generated with homology modeling. These techniques do not require the structures of related complexes to model the association between targets proteins. Consequently, template-free approaches provide a higher coverage in large-scale applications focusing on the construction and analysis of PPI networks. Although template-free modeling is often applied to a pair of proteins known to interact with one another, several studies have successfully employed the exhaustive rigid-body protein docking and post-docking analysis to predict PPIs and PPI networks [17–19]. For instance, a docking experiment comparing the distribution of docking scores collected for proteins known to interact to those between putatively non-interacting proteins was reported [20].

Another study attempted to predict the protein-protein interaction network of the bacterial chemotaxis signaling pathway using an all-to-all docking approach [21]. Here, two docking tools, MEGADOCK [18] and ZDOCK [22], were employed to conduct rigid-body docking of all possible combinations of 101 proteins belonging to 13 families, which are known to be part of the chemotaxis signaling pathway. Based on a previous observation that the decoys of interacting proteins form dense clusters as opposed to the lack of dense clusters formed by non-interacting proteins [17, 18], clustering high-scoring decoys was used to evaluate protein binding affinity and to predict the PPI network. Encouragingly, combining positive predictions from both docking tools correctly identified almost all core-signaling interactions in bacterial chemotaxis. Although the aforementioned methods were shown to discriminate true protein interactions from likely non-interacting pairs, the native complexes of interacting proteins have not been recovered mainly due to an insufficient ranking accuracy of docking algorithms. Further, the reported benchmarking calculations conducted using relatively small datasets of experimental structures may not be indicative of the performance of the proteome-scale identification of molecular interactions.

In that regard, we developed a new approach to discover and model PPIs across proteomes employing an exhaustive all-to-all docking strategy. This pipeline comprises six major steps including protein threading and homology modelling, the prediction of binding interfaces, a rigid body docking, the flexible refinement and scoring of the modeled interfaces, and a series of function annotation filters. Our approach was carefully benchmarked on a large and representative dataset of experimental structures and computer-generated models of target proteins. In order to demonstrate its utility in large-scale projects, we modeled dimer structures and predicted PPIs across the proteome of *Escherichia coli*. Interaction data generated for *E. coli* is primed for experimental validation and further computational analyses. In addition, we validated our method against the human immune disease pathway. Encouragingly, our results demonstrate that protein docking can be used not only to identify near-native complexes but also to predict interaction partners. Overall, this study shows that combining computational modeling, structural bioinformatics, machine learning, and function annotation provides a powerful methodology for the bottom-up assembly of protein-protein interaction networks.

## Methods

### Datasets

The pipeline to model PPIs is benchmarked on the BM1905 dataset (available at http://www.brylinski.org/content/efindsiteppi-datasets), which was previously compiled to evaluate the accuracy of interface residue prediction and the re-ranking of docked models [23, 24]. This dataset contains experimental target structures (BM1905C) as well as high-quality computer-generated models (BM1905H). The quality of monomer models was assessed by the root-mean-square deviation (RMSD) and the Template Modeling score (TM-score) [25]. The latter ranges from 0 to 1 with values >0.4 indicating a significant structural similarity to the native conformation. BM1905H comprises models whose mean Cα-RMSD is 6.94 Å ±4.61 and mean TM-score is 0.72 ± 0.15.

The algorithm to predict binary interactions is trained and validated against a non-redundant and representative dataset of 18,162 protein dimers selected from the PDB. First, all dimers having at least 20 interface residues were categorized as either homo-dimers whose individual chains share at least 85% sequence identity or hetero-dimers when the sequence identity was below 85%. Next, each subset was clustered with CD-HIT [26] at 80% sequence identity. Finally, redundant dimers that have similar interfaces with the Matthews correlation coefficient (MCC) calculated over interface residues of >0.5 were removed from each cluster. This procedure resulted in a set of 14,944 homodimers (HOM14944) and a set of 3,519 heterodimers (HET3519). In addition, the algorithm to predict binary interactions is tested on 1,688 non-interacting protein pairs derived from the Negatome 2.0 database [27]. Computer models of individual proteins in Negatome 2.0 were built with Modeller [28] using templates identified by *e*Thread [29], followed by a high-resolution structure refinement with ModRefiner [30].

The developed pipeline to predict PPI networks is validated using *E. coli* as a model organism. Protein interaction data for *E. coli* consisting of 13,374 known interactions formed by 2,994 bacterial proteins were downloaded from the Database of Interacting Proteins (DIP) [31] in March 2016. We removed from the original dataset redundant proteins as well as those targets longer than 600 residues, which may be difficult to model with threading, and shorter than 50 residues because these molecules are likely peptides. The final *E. coli* dataset consists of 2,300 proteins forming 6,341 interactions. DIP provides the sequences of interacting proteins, therefore, we constructed monomer structures with Modeller [28] using templates identified by *e*Thread [29], followed by a high-resolution structure refinement with ModRefiner [30].

Finally, the protocol to predict and model protein interactions is validated against the human immune disease pathway associated with the Toll-Like Receptor (TLR) signaling cascade. Information on proteins involved in this pathway as well as experimentally detected interactions were obtained from the Reactome database [32] in June 2016. The human immune pathway comprises 26 proteins connected through 112 interactions; protein monomer structures are constructed with the same protocol as that used to model DIP proteins.

### Protein docking, ranking and refinement

For a given pair of protein targets, a collection of docking solutions is generated with the FFT-based rigid body docking program ZDOCK version 3.02 [33]. We use the default parameters to exhaustively search the 3D grid space around the receptor by rotating and translating the ligand. Subsequently, the top 2,000 conformations reported by ZDOCK are re-ranked with *e*Rank^PPI^ [23], a recently developed algorithm to identify near-native conformations from the high-scoring hits. The scoring function implemented in *e*Rank^PPI^ employs multiple features including residue-level interface probability estimates, protein docking potentials, and energy-based scores. Surface residues in target receptors are annotated with interface probability estimates by *e*FindSite^PPI^ [24], a structure/evolution-based approach to detect interface residues. *e*FindSite^PPI^ builds on a strong conservation of the location and geometry of binding sites in evolutionarily related dimers and employs meta-threading, structural alignments, and machine learning to predict interfacial residues for a target protein. The top 10 models selected by *e*Rank^PPI^ are finally subjected to a flexible refinement with FiberDock [34]. FiberDock mimics the induced fit by accounting for both side-chain and backbone flexibility. The side-chain flexibility is modeled using a rotamer library, whereas a normal mode procedure is used to model the backbone flexibility.

### Assessing the quality of protein complex models

The accuracy of dimer models is primarily assessed with iAlign [35] against experimental complex structures retrieved from the PDB. iAlign evaluates the quality of structural models with the Interface Similarity score (IS-score) combining Cartesian distances with the overlap of interfacial contact patterns [36]. IS-score ranges from 0 to 1 with values greater than 0.210, 0.311 and 0.473 indicating a statistically significant interface similarity at *p*-values of 10^-2^, 10^-5^ and 10^-10^, respectively. In addition, the quality of dimer models is assessed with iRMSD, a standard evaluation measure in the Critical Assessment of PRedicted Interactions (CAPRI) [37] and the Pairwise Contact Score (PCS) [23]. iRMSD is the interfacial Cα-RMSD between ligands in the predicted and experimental complexes upon the superposition of receptor structures. In iRMSD calculations, interface residues are defined as those having at least one atom within 10 Å from any atom in the other protein chain. The PCS employs the Matthews correlation coefficient to evaluate the overlap between predicted and the actual interfacial contacts; it ranges from about 0 (random prediction) to 1 (perfect prediction). The docking success rate is defined as the percentage of targets for which at least one correct model is ranked within the top 10 conformations. The acceptance criteria for correct predictions are an iRMSD of ≤2.5 Å and a PCS of ≥0.65 for experimental structures, and an iRMSD of ≤8.5 Å and a PCS of ≥0.30 for computer-generated models, as described in [23].

### Protein-protein interaction prediction with supervised learning

The scoring function to identify biologically relevant assemblies was trained and cross-validated against the HET3519 dataset of experimental hetero-dimers used as positives and a simulated dataset of 14,944 likely non-interacting pairs used as negatives. The negative dataset was constructed by randomly swapping ligands within the HOM14944 dataset. Since HOM14944 proteins share less than 80% sequence identity, this procedure resulted in a random set of hetero-dimers referred to as RND14944. Uniformly choosing random protein pairs excluding experimental interactions produces an unbiased estimate of the distribution of negatives in the prediction of protein-protein interactions [38]. Hence, this procedure is a common practice to generate negative datasets containing at most a negligible fraction of interacting proteins [39–41]. FiberDock calculates several binding energy scores, including attractive and repulsive van de Waals forces, the atomic contact energy, partial electrostatics, hydrogen and disulfide bonds, π-stacking, and aliphatic interactions. These scores were used as a feature vector to train a Random Forest Classifier (RFC) returning a single probabilistic score to assess whether two interacting proteins are biologically relevant. The machine learning model was 10-fold cross-validated against the positive set HET3519 and the negative set RND14944.

### Annotation filters

Positive predictions are further subjected to filtering with Gene Ontology (GO) terms. GO is a hierarchically organized database providing a controlled vocabulary to characterize gene products, divided into three sub-ontologies: cellular component (CC), biological process (BP) and molecular function (MF) [42]. Here, we use GO slims, which are cut-down versions of the GO ontologies without the detail of the specific fine grained terms. GO slims were extracted from the PANTHER classification system [43], whereas annotations for *E. coli* proteins were obtained from the EcoCyc database [44] in May 2016. We tested whether CC, BP and MF slims can be used to refine prediction results by considering proteins localized in the same cellular component, assigned to the same biological process, and having different molecular functions.

### Performance evaluation metrics

PPI prediction is assessed using standard evaluation metrics for classification problems:

True positive rate:

1 $$ T P R=\frac{TP}{TP+ FN} $$

False positive rate:

2 $$ F P R=\frac{FP}{FP+ TN} $$

Accuracy:

3 $$ A C C=\frac{TP+ TN}{TP+ FP+ TN+ FN} $$

Matthews correlation coefficient:

4 $$ M C C=\frac{TP\times TN- FP\times FN}{\sqrt{\left( TP+ FP\right)\left( TP+ TN\right)\left( FP+ FN\right)\left( TN+ FN\right)}} $$

where *TP* (True Positives), *FN* (False Negatives) and *FP* (False Positives) are the number of correctly predicted, under-, and over-predicted PPIs, respectively. *TN* (True Negatives) is the number of correctly predicted non-interacting partners. The MCC quantifies the strength of the correlation between predicted and actual classes; by heavily penalizing both over- and under-predictions, it provides a convenient assessment measure that balances the sensitivity and specificity.

## Results and discussion

**Fig. 1:**
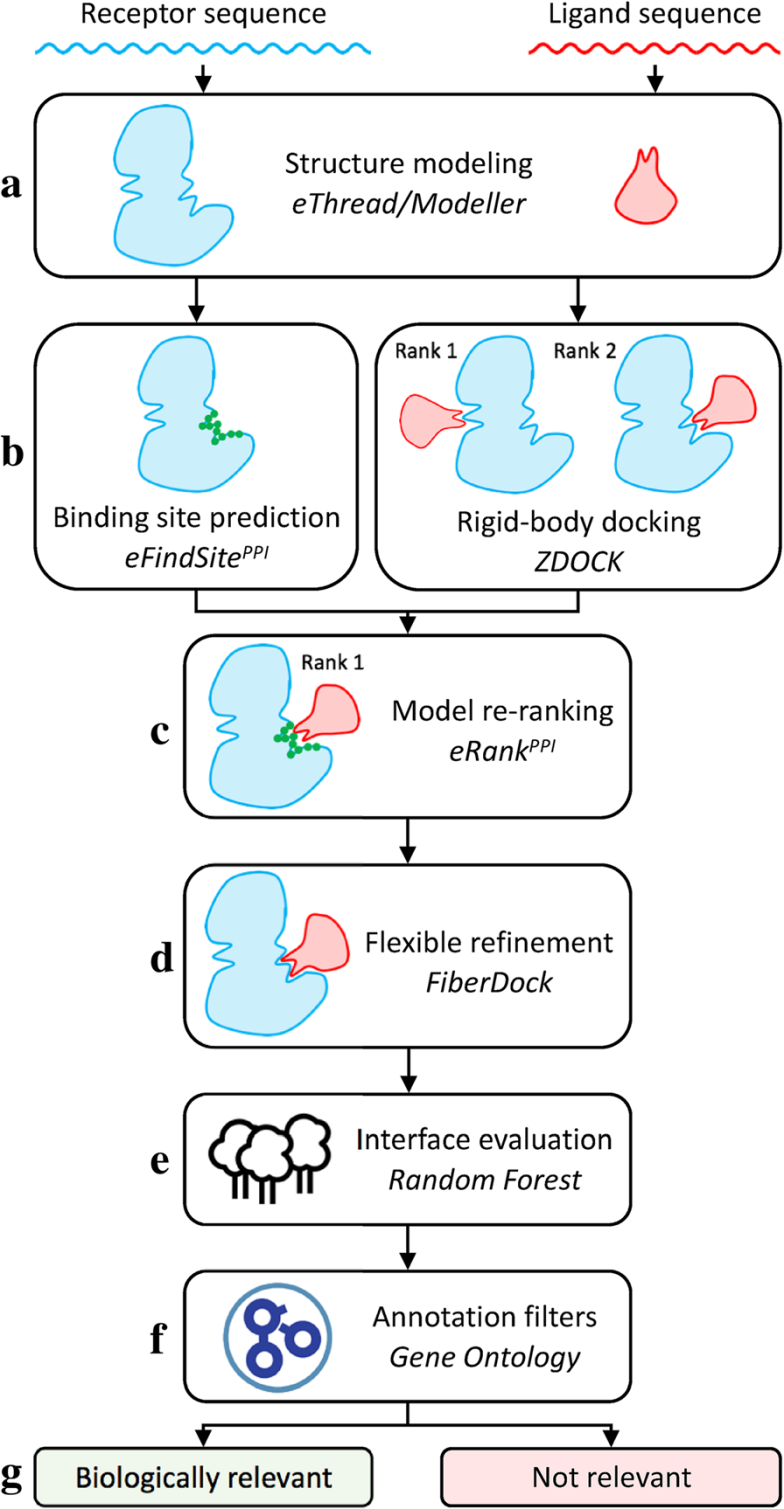
Flowchart of the across-proteome modeling of dimer structures and the prediction of protein-protein interactions. **a** Query protein structures are first built with homology modeling. **b** Subsequently, a binding site is identified in the receptor and initial dimer models are generated through rigid body docking. **c** Initial models are then re-ranked by *e*Rank^PPI^ taking into account the binding site information and (**d**) subjected to a flexible refinement. **e** Machine learning followed by (**f**) annotation filters are finally employed to identify biologically relevant protein assemblies (**g**)

The goal of this study was to develop and test a new protocol to model putative protein complex structures across proteomes that can subsequently be used to assemble protein-protein interaction networks. The modeling procedure for a pair of proteins is presented in Fig. 1. The construction of a hetero-dimer starts with the prediction of 3D structures of individual monomer chains using *e*Thread and Modeller (Fig. 1a). Here, the larger monomer is the receptor and the smaller monomer is the ligand; the size is proportional to the number of amino acid residues. Subsequently, *e*FindSite^PPI^ is employed to predict a protein binding site in the receptor structure and, simultaneously, a rigid-body docking of the ligand to the receptor is performed with ZDOCK (Fig. 1b). In the next step, docking conformations are filtered and re-ranked with *e*Rank^PPI^ utilizing the binding interface predicted by *e*FindSite^PPI^ (Fig. 1c). The identified putative dimers are then subjected to a flexible refinement with FiberDock (Fig. 1d) followed by the evaluation of binding energies with the RFC in order to select the final model (Fig. 1e). A probability score reported by the RFC is used together with annotation filters according to Gene Ontology terms (Fig. 1f) to make the final decision whether or not the constructed dimer is biologically relevant (Fig. 1g).

Although the comprehensive benchmarks of *e*FindSite^PPI^ and *e*Rank^PPI^ have been already reported [23, 24], we found that a flexible refinement improves the accuracy of dimers assembled from experimental as well as computer-generated monomer structures. In addition, using machine learning to evaluate the refined interfaces is shown to reliably detect biologically relevant protein complexes. Finally, we demonstrate that annotation filters can successfully be employed in genome-wide projects to further refine the classification results and more accurately identify putative pairs of interacting proteins.

### Sampling and scoring in template-free docking

In this work, the structures of protein complexes are modeled via a protocol utilizing template-free docking with ZDOCK. Template-free docking consists of two successive tasks, sampling and scoring. Sampling employs a rigid-body search over different rotational-translational degrees of freedom, whereas the purpose of scoring is to rank the sampled poses in order to identify near-native configurations. Consequently, sampling and scoring failures are two major reasons for the lack of success in protein docking. The former are caused by an insufficient sampling, *viz*. near-native conformations are not generated by a sampling algorithm, therefore, reliable dimer models cannot be constructed. These errors can frequently be corrected simply by increasing the sampling exhaustiveness. Scoring failures are unsuccessful docking calculations, in which at least one near-native conformation is generated, however, it is not selected by a scoring function as a feasible solution; correcting these errors is more challenging compared to sampling failures. *e*Rank^PPI^ was developed specifically to address scoring failures by improving the accuracy of dimer ranking in protein docking [23].

**Fig. 2:**
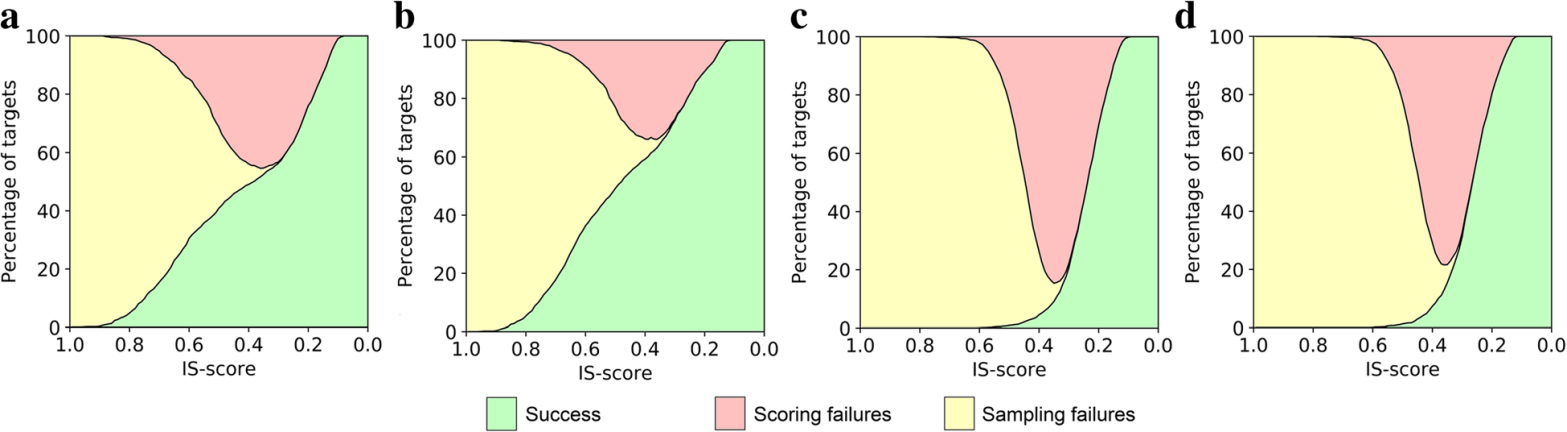
Analysis of success and failure rates in protein docking. Spectrum plots are constructed for (**a**, **b**) crystal monomer structures and (**c**, **d**) protein models. Successful docking cases shown in *green* correspond to those predictions for which at least one native-like configuration with an IS-score greater than a value display on the *x*-axis is ranked within the top 10 poses by (**a**, **c**) ZDOCK and (**b**, **d**) *e*Rank^PPI^. The remaining cases represent two types of docking failures. Scoring failures shown in red correspond to those predictions in which at least one native-like configuration is present in a set of 2,000 dimer models, however, it was not ranked within the top 10 poses. Sampling failures shown in yellow correspond to the remaining cases for which no native-like configurations have been generated

Here, we assess docking success rates, sampling and scoring failures for crystal structures as well as computer-generated models for the BM1905 dataset. The results are shown as IS-score spectrum plots in Fig. 2. For instance, at an IS-score of 0.210 corresponding to a *p*-value of 10^-2^, the success rate of ZDOCK against crystal structures is 73.4%, with the remaining 26.6% cases classified as scoring failures (Fig. 2a). Re-ranking of the docked poses with *e*Rank^PPI^ increases the success rate to 88.1%, decreasing the rate of scoring failures to only 11.9% (Fig. 2b). For computer-generated models, the success rates (scoring failures) are 64.4% (35.6%) for ZDOCK and 71.9% (28.1%) for *e*Rank^PPI^ (Fig. 2c and d, respectively). Note that the lack of sampling failures at an IS-score of 0.210 suggests that rigid-body docking successfully samples the conformational space of dimers assembled with experimental as well as computer-generated models of monomer proteins. Sampling failures come into sight only at higher IS-score values, for example, conformations with an IS-score of at least 0.473 corresponding to a *p*-value of 10^-10^ are not constructed by ZDOCK for 19.1 and 61.1% of the cases when experimental monomer structures and computer-generated models are used, respectively. However, one should keep in mind that the models of individual monomers may already contain significant inaccuracies, thus interfaces highly similar to those in experimental structures simply cannot be constructed by rigid-body docking. Overall, this analysis shows that scoring failures are responsible for the majority of unsuccessful docking calculations and that *e*Rank^PPI^ improves the success rate by reducing the number of scoring failures by 14.7% for crystal structures and 7.5% for protein models.

### Dimers constructed from experimental monomer structures

**Fig. 3:**
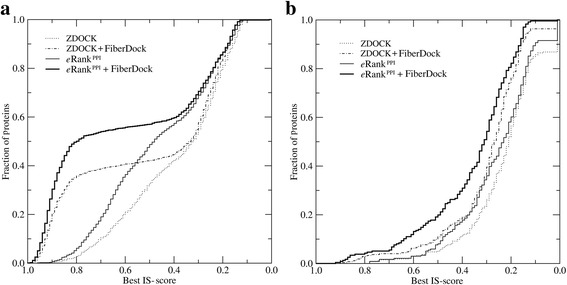
Performance of ZDOCK, *e*Rank^PPI^ and FiberDock on the BM1905 dataset. Dimer complexes are constructed using (**a**) experimentally solved monomer structures (BM1905C) and (**b**) computer generated monomer models (BM1905H). The results are presented as the cumulative fraction of proteins with the IS-score between predicted and experimental complex structures larger than or equal to the value displayed on the *x*-axis

Interface quality in the modeled dimer structures is assessed in Fig. 3 by the distribution of IS-scores [36] across the BM1905 dataset. Figure 3a shows the accuracy of complex models constructed from experimental monomeric structures with ZDOCK alone, ZDOCK followed by FiberDock, eRank^PPI^, and *e*Rank^PPI^ followed by FiberDock. For each receptor-ligand pair, we first selected the top 10 highest scoring ZDOCK models and picked the model with the best IS-score. At least one model with a statistically highly significant IS-score of 0.473 is found in 34.9% of the cases. This percentage increases to 42.4% when the initial dimers are refined by FiberDock. Next, we re-ranked the top 2,000 models from ZDOCK with *e*Rank^PPI^ in order to more reliably identify near-native structures. Encouragingly, in 50.5% of the cases, at least one model having an IS-score higher than 0.473 is now found within the top 10 dimers re-ranked by *e*Rank^PPI^. Further refinement with FiberDock increases this fraction to as high as 57.5%. In addition to the IS-score, Table 1 shows that success rates measured with iRMSD as well as PCS increase when *e*Rank^PPI^ and FiberDock are included in the modeling protocol.

**Table 1:** Comparison of the success rates for protein dimers assembled from the crystal structures and computer-generated models of monomers

Protocol	Crystal structures		Protein models	
	*iRMSD ≤2.5 Å*	*PCS ≥0.65*	*iRMSD ≤8.5 Å*	*PCS ≥0.30*
ZDOCK	51.5%	52.1%	28.1%	23.2%
ZDOCK + *e*Rank^PPI^	58.3%	59.6%	43.7%	39.3%
ZDOCK + *e*Rank^PPI^ + FiberDock	72.8%	73.2%	52.4%	48.7%

The acceptance criteria for correct predictions are an iRMSD of ≤2.5 Å and PCS ≥0.65 for crystal structures, and an iRMSD of ≤8.5 Å and PCS ≥0.30 for protein models. The best of top 10 dimer models is considered

Altogether, *e*Rank^PPI^ and FiberDock generate the most accurate dimers in these benchmarking calculations. Figure 3a and Table 1 show that re-ranking with *e*Rank^PPI^ places more near-native structures within the top-ranked models compared to ZDOCK, which is in accordance with our previous studies [23] reporting ~10% improvement in the success rate. In general, the refinement by FiberDock considering both backbone and sidechain flexibility consistently improves the model accuracy, however, the improvement clearly depends on the quality of the top-ranked dimers. Most significant improvement for models selected by *e*Rank^PPI^ is achieved when the IS-score of the initial dimers is in the range of 0.4-0.8.

### Dimers constructed from computer-generated monomer structures

The unavailability of experimentally determined structures for a vast majority of gene products necessitates using computer-generated models for genome-wide determination of PPIs. On that account, we investigate how protein docking, and dimer re-ranking and refinement are affected when computer-generated models are used instead of experimental structures. Figure 3b shows the accuracy of dimer models constructed using four protocols described above. Since monomers are weakly homologous models containing structural inaccuracies, the modeling results are evaluated with a lower, yet still statistically significant IS-score threshold of 0.311. We find that in 22.3 and 31.0% of the cases, at least one model with an IS-score of ≥0.311 is found within the top 10 conformations ranked by ZDOCK and *e*Rank^PPI^, respectively. Furthermore, a flexible refinement with FiberDock increases the percentage of successful cases to 32.2% for ZDOCK and to 48.7% for *e*Rank^PPI^. Table 1 shows that similar results are obtained with the iRMSD and PCS used to measure the success rate. Therefore, not only dimer models re-ranked by *e*Rank^PPI^ and additionally refined by FiberDock are the most accurate, but also the refinement procedure yields better improvements for *e*Rank^PPI^ compared to ZDOCK. Despite the fact that protein docking using weakly homologous monomer structures is a difficult task and the dimer accuracy cannot be expected to be higher than the accuracy of the monomers, our analysis demonstrates that, in many cases, using a protocol combining *e*Rank^PPI^ and FiberDock constructs reliable complexes as assessed by the IS-score, iRMSD, and PCS.

### Predicting biologically relevant interactions

**Fig. 4:**
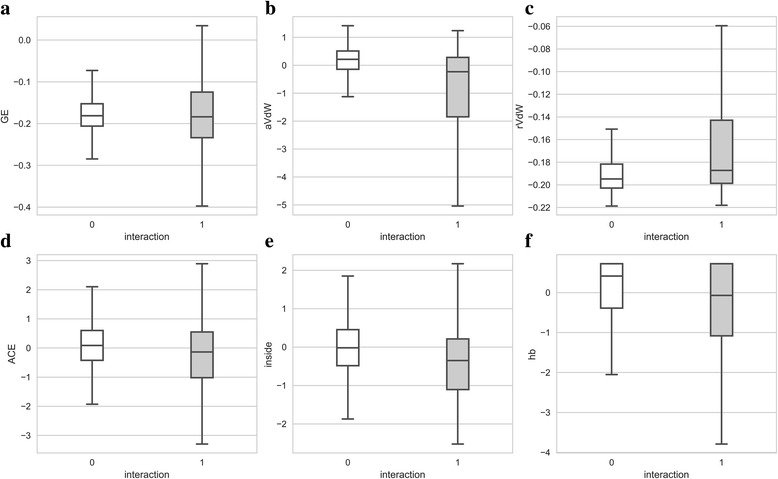
Distribution of various components to the binding energy calculated with FiberDock. Negative pairs from the RND14944 dataset and positive pairs from the HET3519 dataset are shown as *white* and *gray* boxes, respectively. The following normalized (*Z*-score) energy terms are shown: (**a**) global energy, (**b**) attractive van der Waals potential, (**c**) repulsive van der Waals potential, (**d**) atomic contact energy, (**e**) internal energy, and (**f**) hydrogen bond potential. Boxes end at quartiles Q_1_ and Q_3_ and a horizontal line in each box is the median. Whiskers point at the farthest points that are within 1.5 of the interquartile range

Macromolecular complexes are stabilized by a variety of interactions including solvation effects, changes in the internal energy upon binding, electrostatics, van der Waals interactions, hydrogen bonds, π-stacking, and hydrophobic contacts across the interface. These interactions are prevalently found in the crystal structures of protein assemblies deposited in the PDB. Given that protein crystals mimic the actual interactions in an aqueous solution, biologically relevant complex structures can be predicted based on these contributions to the binding energy. Figure 4 shows the distribution of various energy terms calculated by FiberDock for the positive dataset HET3519 and the negative dataset RND14944. Note a clear distinction in the distribution of most energies between interacting and non-interacting protein pairs suggesting that these scores can be utilized to identify biologically relevant interactions. For example, the median attractive (repulsive) van der Waals energy is -0.230 (-0.187) and 0.214 (-0.195) for interacting and non-interacting pairs, respectively. Another highly discriminatory term is the hydrogen bond energy with the median value of -0.068 for interacting and 0.418 for non-interacting pairs, which is consistent with other studies reporting that the hydrogen bond potential greatly improves the recognition of correctly docked protein-protein complexes from large sets of alternative structures [45].

**Fig. 5:**
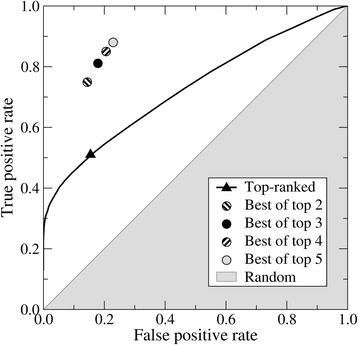
Receiver operating characteristic (ROC) plot evaluating the accuracy of the prediction of biologically relevant PPIs for the HET3519 and RND14944 datasets. The solid line corresponds to the performance of a Random Forest Classifier employing the top-ranked models with the black triangle pointing out the highest accuracy. Circles represent the performance achieved by considering the top 2, 3, 4 and 5 ranked models for each target complex. The gray area shows the performance of a random classifier

Next, we combine various interactions at the interface for the top 3 refined models in order to evaluate the complex stability and to predict whether the interaction is biologically relevant or not. Specifically, the RFC is employed to estimate a probability that a given complex model represents a true interaction. Figure 5 shows a receiver operating characteristic (ROC) plot evaluating the performance of a classifier separating true interactions within the HET3519 dataset from negative pairs present in the RND14944 dataset. Using the top-ranked model, the area under the curve for the prediction of biologically relevant interactions is 0.72. The probability threshold of 0.13 (a solid triangle in Fig. 5) maximizes the MCC to a value of 0.43 at a true positive rate of 0.51 and a false positive rate of 0.14. Essentially, this threshold corresponds to a point in the ROC space farthest from the diagonal representing the performance of a random classifier (gray area in Fig. 5).

Next, we improved the classification procedure by employing up to top 5 ranked models constructed for a given pair of receptor and ligand proteins. A pair is predicted to represent a true interaction if a positive predictive score is greater than the optimized probability threshold of 0.13 for at least one out of top *n* models. Table 2 shows that this strategy indeed enhances the discriminatory power. Considering the top 3 models maximizes the MCC to a value of 0.61 with a true positive rate of 0.81 and a false positive rate of 0.19 (a solid circle in Fig. 5). Finally, we independently test our classification protocol against the Negatome 2.0 database, which provides a collection of protein pairs unlikely to physically interact with each other [27]. We obtained a false positive rate of 0.23, i.e. 23% of non-interacting pairs included in Negatome 2.0 are predicted as interacting proteins. This false positive rate is similar to that calculated for the HET3519 and RND14944 datasets suggesting that the RFC classifier is robust and its performance is independent on the validation dataset. Overall, the classifier performance is sufficiently high to be applicable at a proteome scale.

**Table 2:** Accuracy of the prediction of biologically relevant PPIs for the HET3519 and RND14944 datasets

Number of models	MCC	TPR	FPR
1	0.43	0.53	0.11
2	0.58	0.74	0.14
3	0.61	0.81	0.19
4	0.58	0.85	0.20
5	0.58	0.88	0.22

Here, we consider up to top 5 ranked models constructed for a given pair of receptor and ligand proteins

*MCC* Matthews correlation coefficient, *TPR* true positive rate, *FPR* false positive rate

### Modeling protein-protein complex structures for *E. coli*

All-against-all docking of 2,300 proteins in *E. coli* produced 2,643,850 possible binary PPIs with 3 putative dimer models generated for each unique receptor-ligand pair, totaling 7,931,550 3D complex structures of bacterial proteins. Applying the RFC trained on the HET3519 and RND14944 datasets predicted 425,412 biologically relevant interactions corresponding to 18.2% of all possible PPIs (Additional file 1). Note that although the experimentally covered PPI space provided by DIP [31] is very limited with only 6,341 validated interactions, our structure-based pipeline correctly identified 3,930 (62%) of these true PPIs. According to the BioGRID Database Statistics, an estimated number of 164,717 non-redundant interactions are present in *E. coli*, suggesting that that additional filters are required to further refine the set of predicted interactions. On that account, we added annotation filters from Gene Ontology to support the identification of biologically relevant dimers constructed for the *E. coli* proteome.

### Integrating structure-based prediction with Gene Ontology

First, we tested whether CC, BP and MF slims can be used as filters to identify interacting proteins by comparing GO annotations in positive and negative protein pairs. Here, the positive set contains known protein interactions according to the DIP database, whereas the negative set is compiled by randomly pairing *E. coli* proteins included in the DIP database. Those protein pairs having at least one common GO slim pass the annotation filter. About 82% of positives pass the CC filter that requires two proteins to co-localize in order to form a physical interaction. In contrast, only 58% of negatives are located in the same cellular component. Further, as many as 93% of positives are part of the same biological process, whereas 66% of negatives pass the BP filter. These results are in line with previous studies demonstrating that proteins localized in the same cellular compartment are more likely to interact than those residing in spatially distant compartments [46, 47]. Similarly, proteins involved in the same biological process have on average a higher chance to interact compared to molecules functioning in different biological processes. Thus, both CC and BP filters retain the majority of true interactions and reject a number of non-interacting protein pairs leading to a better classification performance. In contrast, molecular function cannot be used to improve the identification of biologically relevant interactions because a similar percentage of positives (48%) and negatives (52%) pass the MF filter. To further corroborate these results, we applied both CC and BP filters to the HET3519 and RND14944 datasets. Encouragingly, as many as 91 and 93% of HET3519 complexes passed CC and BP filters, respectively. In contrast, significantly fewer pairs from the random dataset RND14944 passed CC (63%) and BP (44%) filters. The discriminatory performance of GO filters applied to HET3519 and RND14944 is consistent with that obtained for the *E. coli* dataset.

### Assembly and analysis of PPI network in E. coli

In order to assemble the network of protein-protein interactions in *E. coli*, we first applied the CC filter to 425,412 putative hetero-dimers identified by the RFC bringing this number down to 253,230 interactions between proteins localized in the same cellular compartment. Next, we selected only those protein pairs involved in the same biological process further reducing the number of putative hetero-dimers to 81,280. Although the BP filter is highly sensitive correctly identifying 93% of true interactions, this significant reduction of the number of positive predictions is mainly attributed to the fact that BP annotations are available for only 1,294 out of 2,300 proteins. Combining structure-based prediction of PPIs with both annotation filters results in 61,913 biologically relevant interactions. Note that GO filters are frequently employed to automatically refine large sets of protein interactions. For instance, the *F*-measure assessing the accuracy of PPI prediction for the bacterial chemotaxis signaling pathway increased from 0.52 to 0.69 when the protein localization was taken into consideration [21]. Our final set of protein interactions with confidently modeled dimer conformations provide a tremendous source of structural data relating to the network of protein-protein interactions in *E. coli*.

Subsequently, we investigated several properties of the PPI network constructed for *E. coli* in comparison with a random network comprising the same number of nodes and edges. The only difference between the predicted and random networks is that the latter is built on interactions randomly assigned to pairs of proteins. For the PPI network predicted for *E. coli* by the structure-based approach, the degree, diameter, and clustering coefficient [48] are 110.5, 6, and 0.30, respectively. Although the random network has a similar degree of 111.4, its diameter is 3 and the clustering coefficient is only 0.11. This analysis reveals that the global topology of the constructed network significantly differs from that of a random network. Specifically, the predicted PPIs tend to cluster together forming functional units around highly connected hubs, whereas PPIs are distributed more uniformly in a random network. In order to further corroborate these findings, we constructed a PPI network from experimental interactions included in the DIP database and the corresponding random network having the same number of nodes and edges. Here the degree, diameter and clustering coefficient calculated for the DIP (random) network are 6.9 (6.8), 12 (7), and 0.08 (0.004), respectively. The differences between the network predicted by a structure-based approach and that built on interaction data from DIP result from the incompleteness of the latter, i.e. the DIP network is sparse, having about 17 times less connections per node than the predicted network. Nonetheless, the deviations of both networks from their random counterparts are qualitatively similar showing a notable tendency to form clusters and sub-networks.

**Fig. 6:**
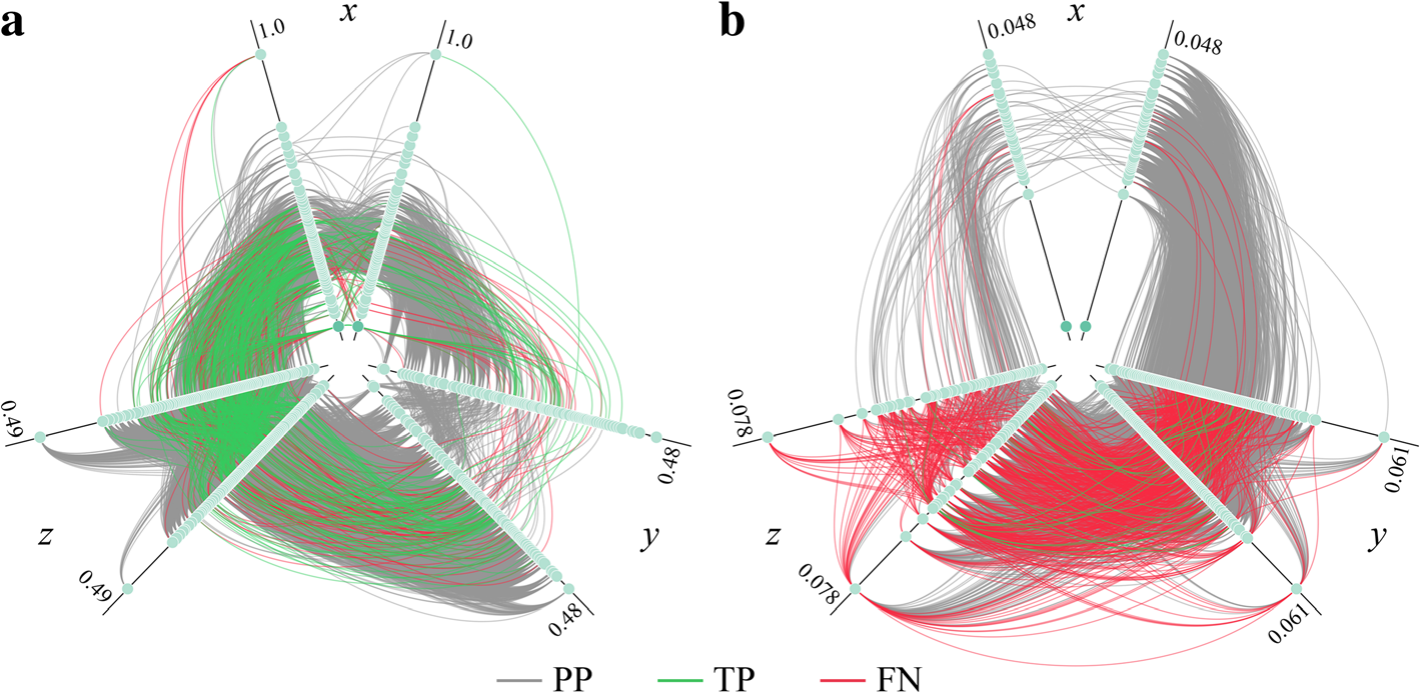
Hive plots of PPI networks for the proteome of *E. coli*. Turquoise circles (nodes) represent individual proteins connected by interactions (edges). Three types of interactions are denoted by edges in different colors, positive predictions are *gray*, true positives (predicted interactions also present in the DIP database) are *green*, and false negatives (DIP interactions that are not predicted) are *red*. **a** Network constructed by modeling the structures of hetero-dimer complexes followed by the classification of interfaces with machine learning. **b** Random network comprising the same number of nodes and edges as the structure-based network, however, with interactions randomly assigned to pairs of nodes. *E. coli* proteins are assigned to three axes based on their degree *d*, low-degree (*d* <50) on the *x*-axis, medium-degree (50 ≤ *d* ≤80) on the *y*-axis, and high-degree (*d* >80) on the *z*-axis. Each axis is then split into two identical axes in order to show interactions within each group. Further, nodes on the axes are sorted by the increasing clustering coefficient *c* with the maximum value of *c* shown next to each axis (note the significant scale difference between **a** and **b**)

Figure 6 shows hive plots [49] generated for the predicted (Fig. 6a) and random (Fig. 6b) networks of PPIs in *E. coli*. In both plots, true positives and false positives with respect to experimentally validated interactions from the DIP database are colored in green and red, respectively. First, the structure-based approach including GO filters correctly identifies the majority of experimental interactions (green lines), whereas these connections are largely missed in the random network (red lines). Second, the axes in both hive plots are sorted by the clustering coefficient of individual nodes and the axis scales in Fig. 6a and b are significantly different. Third, considering the global network topology, the majority of nodes in the random network are assigned to a medium-degree group (*y*-axis) forming extensive connections to themselves as well as to low- (*x*-axis) and high-degree (*z*-axis) groups. In contrast, extensive connections between all groups are present in the network predicted by the modeling of quaternary structures. These hive plots effectively visualize differences between the predicted and random networks described above.

### Examples of dimer models selected from the E. coli network

**Fig. 7:**
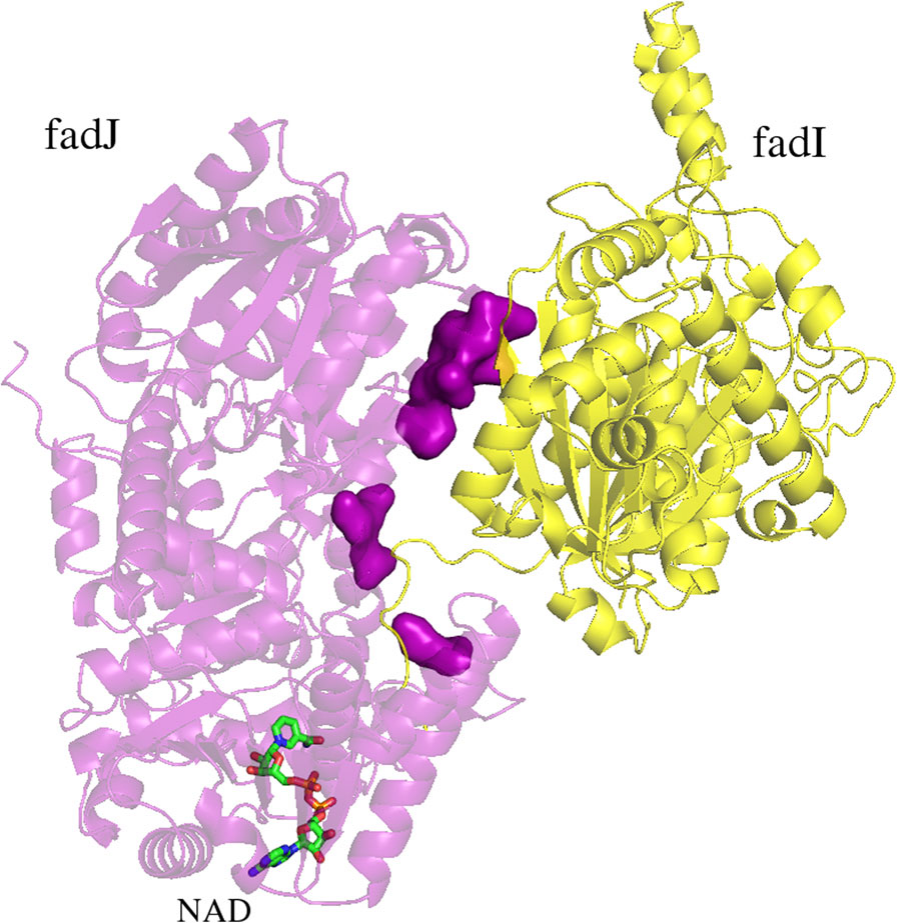
Example of PPI prediction for a hetero-dimer. Cartoon representation of the dimer complex of fadI (*yellow*) and fadJ (*purple*). Interface residues predicted for the receptor are shown as a solid surface. A small molecule ligand (NAD) docked to fadJ is shown as sticks colored by atom type

Since the PPI network for the *E. coli* proteome is assembled by the modeling of interactions between proteins, we discuss a couple of representative examples of the modeled dimer structures. Note that experimentally solved structures are unavailable for these proteins, therefore, the presented molecular assemblies have been constructed solely from the primary sequences of individual monomers. Although monomer models are built on templates whose sequence identity to the target protein is less than 40%, the estimated Global Distance Test (GDT) [50] is greater than 0.7 indicating that these computer-generated structures are highly confident. The first example is a hetero-dimer assembled from fadJ and fadI proteins involved in the fatty acid beta oxidation pathway, which is part of lipid metabolism. This interaction was proposed to increase the efficiency of anaerobic beta-oxidation by favoring substrates of different chain length [51]. Even though there is experimental evidence that these two proteins interact with one another [52], no structural data is available for the individual proteins nor the complex. The modeling procedure developed in this study correctly identified these proteins to be interaction partners with the putative fadJ/fadI hetero-dimer shown in Fig. 7. A protein binding site confidently predicted by *e*FindSite^PPI^ on fadJ comprises 11 residues, out of which 9 are also found at the interface in the modeled fadJ/fadI complex. Moreover, fadJ has a NAD binding domain according to the Pfam database [53]. Interestingly, we were able to not only identify a binding pocket for NAD in the fadJ structure model with *e*FindSite [54], but also to dock a NAD molecule to this pocket using our in-house ligand docking software *e*SimDock [55].

**Fig. 8:**
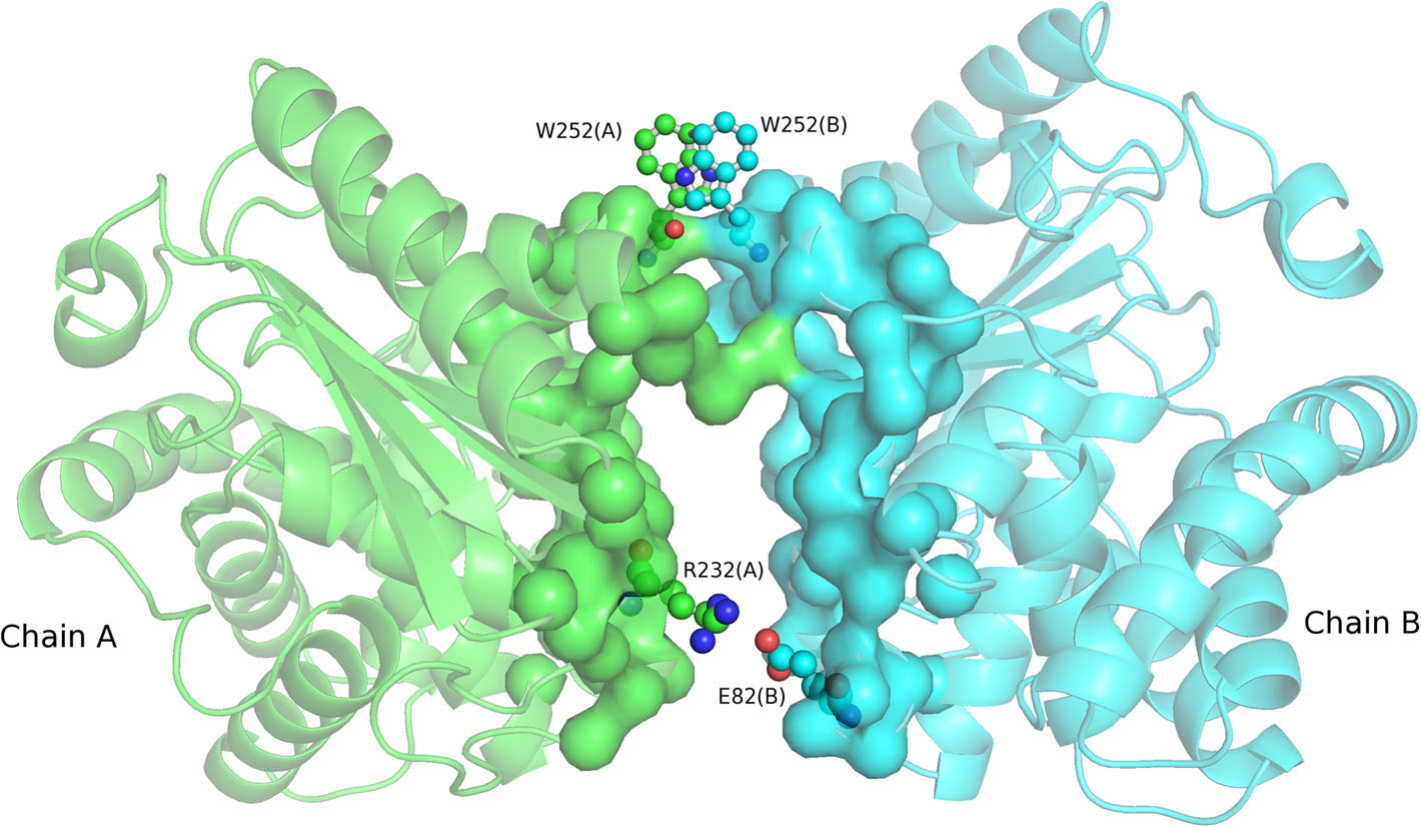
Example of PPI prediction for a homo-dimer. Cartoon representation of the dimer complex of YneH with chains A and B colored in *green* and *blue*, respectively. Protein interfaces predicted for the monomers are shown as a solid surface. Residues predicted to be involved in a salt bridge R32(A)-E28(B) and aromatic contact W525(A)-W525(B) are shown as balls and sticks

The second example is glutaminase 2 (glsA2), an amidohydrolase enzyme responsible for generating glutamate from glutamine, demonstrated to be a self-assembling protein [56]. The GDT of the glsA2 monomer estimated by *e*Thread is 0.78 indicating a confident structure model. Next, we predicted the structure of glsA2 homo-dimer as a symmetric complex shown in Fig. 8. A unique feature of *e*FindSite^PPI^ is that it not only detects interaction sites, but also points out specific molecular interactions that stabilize a putative complex. Molecular interactions predicted by *e*FindSite^PPI^ for glsA2 include a salt bridge between the side chains of R232 (chain A) and E82 (chain B) as well as aromatic contacts between W252 (chain A) and W252 (chain B), which are found in the top-ranked complex model selected by *e*Rank^PPI^.

### Analysis of PPIs in the human immune disease pathway

**Fig. 9:**
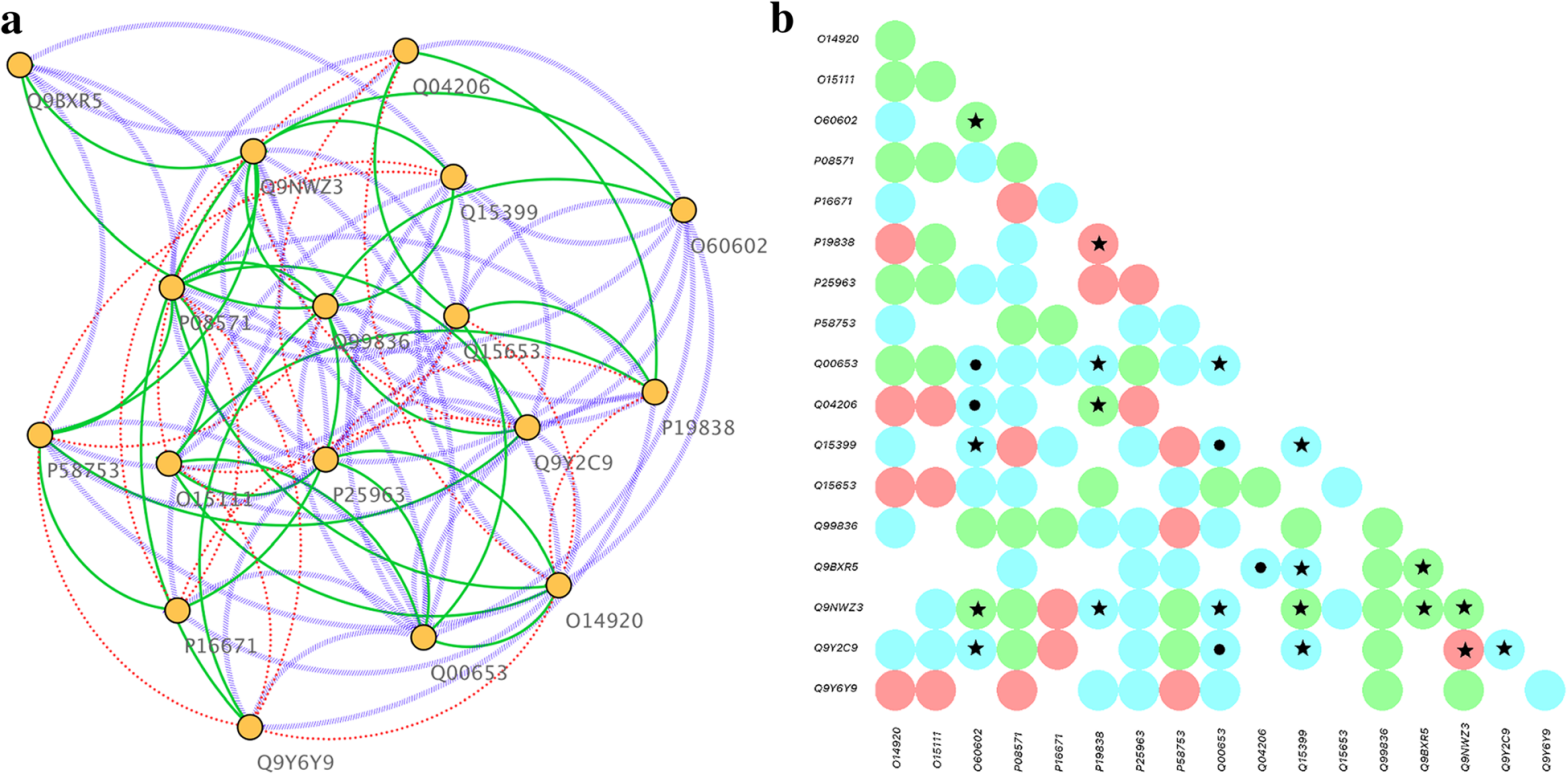
Structure-based prediction of PPIs for the human immune disease pathway. **a** Network diagram of the human immune disease pathway. *Yellow* circles (nodes) represent individual proteins connected by interactions (edges). Three types of interactions are denoted by edges in different colors, positive predictions are *blue*, true positives (predicted interactions also present in the Reactome database) are *green*, and false negatives (interactions from Reactome that are not predicted) are *red*. **b** Matrix of binary interactions including positive predictions (*blue*), true positives (*green*), and false negatives (*red*). Circles marked with a star and a dot show those protein pair that pass and fail the CC filter, respectively. UniProt IDs of proteins involved in this pathway according to the Reactome database are shown in both **a** and **b**

Finally, based on experimental data provided by the Reactome database, we modeled protein complex structures for the human immune disease pathway associated with the TLR signaling cascade. TLRs are sensors of the innate immune system recognizing pathogen-associated molecular patterns [57, 58]. These molecular sensors participate in the first line of defense against invading pathogens by promoting the activation and nuclear translocation of certain transcription factors to induce the secretion of inflammatory cytokines. Out of 26 gene products involved in this pathway, we included the following 17 proteins whose 3D structures have been modeled (estimated GDT values are given in parentheses): P58753 (0.64), Q15399 (0.45), Q9Y2C9 (0.46), P08571 (0.48), P16671 (0.59), O15111 (0.56), O14920 (0.54), Q99836 (0.48), Q9NWZ3 (0.65), O60602 (0.49), Q15653 (0.71), Q00653 (0.32), Q04206 (0.52), P25963 (0.70), P19838 (0.33), Q9BXR5 (0.41), and Q9Y6Y9 (0.77). The remaining 9 structures have not been modeled due to either their large size, the unavailability of reliable templates, or a significant content of transmembrane regions. Although the total number of possible interactions for this dataset is 153, only 58 are confirmed experimentally according to the Reactome database. Figure 9 shows the network structure and a binary interaction matrix for PPIs predicted for this pathway. The structure-based approach predicted a total of 90 unique interactions (dashed blue connections in Fig. 9a) including 38 known interactions (solid green connections in Fig. 9a). Only 20 known interactions have not been predicted by the quaternary structure modeling (dotted red connections in Fig. 9a). Therefore, about two-thirds of true PPIs were correctly recovered by the modeling of the complex structures of proteins involved in the human immune disease pathway. These results are in line with the analysis of the interaction network in *E. coli*, where our protocol correctly identified 62% of known PPIs.

In addition, positive predictions, true positives and false negatives are shown as a binary interaction matrix in Fig. 9b. Here, we also mapped GO Slims for the cellular component to individual proteins in order to improve the PPI prediction accuracy by including function annotation filters. Since GO annotations were available only for 8 proteins, the CC filter was applied to 17 hetero-dimer models constructed by our structure-based approach. Encouragingly, 12 of the predicted complexes passed the CC filter (black stars in Fig. 9b), while only 5 failed (black dots in Fig. 9b). Although, the GO annotation filter can be applied only to a fraction of structure-based predictions for this pathway, it turns out to be quite accurate. Therefore, we expect that new function annotations available in the future will selectively reduce the number of positive predictions leading to more accurate PPI prediction results.

## Conclusions

In this work, we developed a new method combining molecular modeling, structural bioinformatics, machine learning, and functional annotation data to predict PPIs across proteomes. We first comprehensively tested this protocol on representative datasets of experimental structures and computer-generated models of protein dimers and then we applied this methodology to predict PPIs across the proteome of *E. coli* and within the human immune disease pathway. Our results indicate that protein docking supported by evolutionary restraints and machine learning can be used to reliably identify and model biologically relevant protein assemblies. Furthermore, the accuracy of the identification of interaction partners can greatly be improved by including only those protein pairs co-localized in the same cellular compartment and involved in the same biological process. The proposed method can be applied to detect PPIs in other organisms and pathways as well as to construct structure models and estimate the confidence of interactions experimentally identified with high-throughput techniques. Finally, with the growing volume of structural data, experimentally confirmed protein interactions, and functional annotation, we expect the coverage and accuracy of our approach to increase over time.

## Additional file

Additional file 1: A text file containing binary interactions predicted for E. coli proteins. (ZIP 1940 kb)

## Abbreviations

BP: Biological process
CAPRI: Critical Assessment of PRedicted Interactions
CC: Cellular component
DIP: Database of Interacting Proteins
GDT: Global Distance Test
GO: Gene Ontology
IS-score: Interface Similarity score
MCC: Matthews correlation coefficient
MF: molecular function
PCS: Pairwise Contact Score
PDB: Protein Data Bank
PPIs: Protein-protein interactions
RFC: Random Forest Classifier
RMSD: Root-mean-square deviation
ROC: Receiver operating characteristic
TLR: Toll-Like Receptor
TM-score: Template Modeling score
